# Integrating systems thinking and implementation science in biomedical informatics research

**DOI:** 10.1093/jamiaopen/ooag023

**Published:** 2026-03-06

**Authors:** Craig E Kuziemsky, Mustafa Ozkaynak, Kim M Unertl, Saira Haque

**Affiliations:** MacEwan University, Edmonton, Alberta, T5J 4S2, Canada; University of Colorado, Anschutz Medical Campus, Aurora, CO, 80015, United States; Vanderbilt University Medical Center, Nashville, TN, 37203, United States; Pfizer, Inc, 66 Hudson Boulevard East, New York, NY, 10001-2192, United States

**Keywords:** systems thinking, implementation, upstream, interactions

## Abstract

**Objective:**

Implementing biomedical informatics (BMI) tools and applications into clinical and other settings remains a significant challenge. Unintended consequences remain common post-implementation. This paper addresses the above issue by integrating systems thinking and implementation science approaches to support health information technology (HIT) implementation.

**Materials and Methods:**

We reviewed systems thinking and implementation science approaches. We then formally integrated systems thinking and implementation science to better understand system interactions to address implementation issues at the patient, organizational, and broader health system levels.

**Results:**

We developed a four-stage systems-based approach and a set of five pragmatic principles to support systems-based implementation research.

**Discussion:**

Our four-stage approach helps understand the dynamic interactions across system concepts during HIT implementation. Our principles advocate viewing HIT implementation as a “system of systems” rather than as individual entities, using appropriate terminology to describe a system, and using lifecycles and iterative upstream approaches to guide implementation research.

**Conclusion:**

We must draw on the existing body of evidence from the systems thinking and implementation science literature. Our findings build on the strong foundation of systems thinking and implementation science research and practice. We also provide guidance on how to incorporate this knowledge systematically and practically.

## Introduction

Health system pursuits, such as collaborative care delivery, public health and wellness, and patient-centered care, extend beyond individual factors, including technologies, users, policies, or contexts. While taking stock of individual system components in biomedical informatics (BMI) research is necessary and important, health systems are integrated, dynamic, and longitudinal.^1^ BMI research must focus on the relationships and interactions among system components over time. Systems thinking approaches are essential for understanding the dynamic, interrelated components and relationships that support the design and implementation of health information technology (HIT).^2^^,^^3^

Systems thinking approaches have studied interactions between people, process, and technology across settings, including acute, perioperative, and critical care.^3–5^ However, systems thinking is often used descriptively or conceptually without appropriate methodological rigor or alignment with the literature.^6^^,^^7^ Studies often describe a “complex” setting or intervention without defining complexity or situating the study within an appropriate framework, such as the Cynefin framework.^8^ One reason for the ad-hoc and descriptive use of systems thinking is the lack of pragmatic guidance for systems thinking approaches in health systems research.^9^^,^^10^

Implementation science focuses on understanding dynamic systems and processes to incorporate and support the uptake of evidence into practice.^11^ While several implementation science theories and frameworks exist [eg, ^12^], they do not adapt well to dynamic or non-linear systems, resulting in broad solutions that do not always scale down to the diverse contexts where HIT is used.^2^^,^^10^^,^^13^

While systems thinking and implementation science are both used in BMI research,^5^^,^^14–16^ formalized and purposeful integration of the two approaches is less common. Health systems research requires the pursuit of standards for research quality and generative learning that enables pragmatic adaptation of implementation approaches across diverse or changing contexts.^10^ Overall, systems thinking is underutilized in health systems research^17^ while implementation frameworks are often suboptimal^18^ or lack pragmatic details.^19^

The integration of systems thinking and implementation science has been advocated for and pursued in other health disciplines^20–21^. However, it has not been pursued in BMI research. This paper reviews systems thinking and implementation science to provide practical guidance for integrating the two approaches in BMI research. An integrated approach could expand research and theory development in BMI while also encouraging interdisciplinary research with other disciplines.

### Why do we keep putting old wine in new bottles?

Unintended consequences (UICs) occur even with the most carefully designed HIT. Implementation and ongoing use are not just technological, data standard, organizational behaviour, or change management endeavours, but rather are system-based endeavours.^10^^,^^15–16^ We must move beyond trying to prevent UICs or to identify and categorize them after they occur, as integrated systems of people, processes, and technology make it impossible to anticipate all post-implementation outcomes.^22^ Rather, BMI research should focus on the upstream identification of UICs to proactively address them throughout HIT implementation.

Upstream system design approaches begin by defining system needs, priorities, capacity, and existing skills, but must also consider a system’s adaptability as needs or contexts change during downstream processes. Upstream approaches involve defining and using appropriate terminology, such as distinguishing between unintended (not necessarily expected) and unanticipated (unforeseen outcomes) consequences.^23^ Both can be positive and negative. It also requires accuracy with the term “complex.” While definitions of complexity vary across disciplines, a relevant definition for BMI is that complexity involves the degree of interrelations amongst system components.^7^ A greater number of components can make a system complicated but not necessarily complex.^7^ Complex systems differ from simple and complicated systems due to the unpredictable, dynamic nature of interactions among system components.^13^ Other definitions and measurements of complexity exist, for example,^24^ and should be used as appropriate.

Finally, implementation is more than simply installing physical or digital infrastructure such as HIT, Artificial Intelligence, or other digital health tools. It is a dynamic process that aligns technical, social, organizational, and other system components to achieve desired outcomes such as safe, effective, patient-centered care delivery.^25^ HIT implementation is a learning system of systems that is shaped by context and other system components. We must not view downstream outcomes, such as unintended or unanticipated negative consequences, as simply a sign of failure, but rather an opportunity to learn and adapt accordingly.^15^^,^^26^ Health systems are learning systems,^27^ and we should anticipate and plan to redesign or modify specific requirements as the system evolves and we learn more about its properties and how they interact. Similarly, we must move beyond the prevalent find-and-fix approach of reacting to UICs rather than proactively managing them. Systems thinking can support innovative upstream system design approaches that focus on system behaviour over time, enabling an understanding of the dynamic nature of HIT implementation.^2^^,^^3^^,^^28^

### Towards better alignment of implementation science and systems thinking

Systems thinking approaches must have a clear purpose and draw on appropriate methodological guidance.^10^ Relevant theories or methods should be identified and referenced rather than being used as labels or artifacts applied to any BMI study. We define systems by properties such as boundaries, goals, subsystems, skills, capacity, needs, structures, and behaviours, as well as tenets such as non-linearity, emergent behaviours, and simple rules that evolve over time.^2^^,^^3^^,^^7^^,^^10^ Defining and discussing a system using appropriate methods and properties, and properly using terms such as “complexity,” are necessary to enable the replication of findings in systems-based implementation research.

Several types of frameworks and models can support the implementation of HIT. One category is behavioural adoption models from the information systems discipline, such as the Unified Theory of Acceptance and Use of Technology (UTAUT) and the Technology Acceptance Model (TAM).^22^^,^^25^^,^^29^^,^^30^ Another category are evaluation approaches, such as usability testing.^31^ Focused informatics models, such as the Contextual Implementation Model,^32^ are yet another category. Finally, there are dedicated implementation science theories, models, processes, and frameworks.^12^^,^^33^ A common shortcoming of the above approaches is their narrow focus on individual user behaviour or technology and a lack of attention to broader system interactions.^18^^,^^28^ While each of the above approaches has value, we lack systems-based implementation approaches to conceptualize and characterize HIT implementation as a dynamic system of interacting components. It is not our intention to suggest that any existing model or approach is flawed, but rather to advocate for integrated, systems-based perspectives on implementation to help ensure models are fit for purpose. For example, while the Consolidated Framework for Implementation Research (CFIR) acknowledges that the characteristics of individuals, technology, and other system constructs impact implementation, it is limited in its ability to understand the interrelations among these constructs or their evolution.^25^^,^^30^ The Fit between Individuals, Task, and Technology (FITT) framework considers interactions between system components and may be better suited for studying dynamic system interactions.^30^

Upstream system design is about understanding system interactions and evolution over time rather than focusing on individual components at a single moment. System interactions alter the dynamics of a system, leading to unintended or unanticipated consequences, because altering one component can affect other components in unexpected ways.^14^^,^^28^ While implementation science offers methods for incorporating evidence into practice through a set of stages to guide implementation,^33^ dynamic health systems are not conducive to overly rigid or linear models. Dynamic systems are evolving and must be accounted for during implementation. Systems-based modelling approaches^14^^,^^34^ could extend or supplement existing evaluation approaches to account for the dynamic interactions that characterize health systems. Upstream system-based implementation approaches can help minimize unintended consequences (by being proactive) and support ongoing monitoring to prevent downstream unintended consequences (resulting from system adaptation).

**Figure 1.**
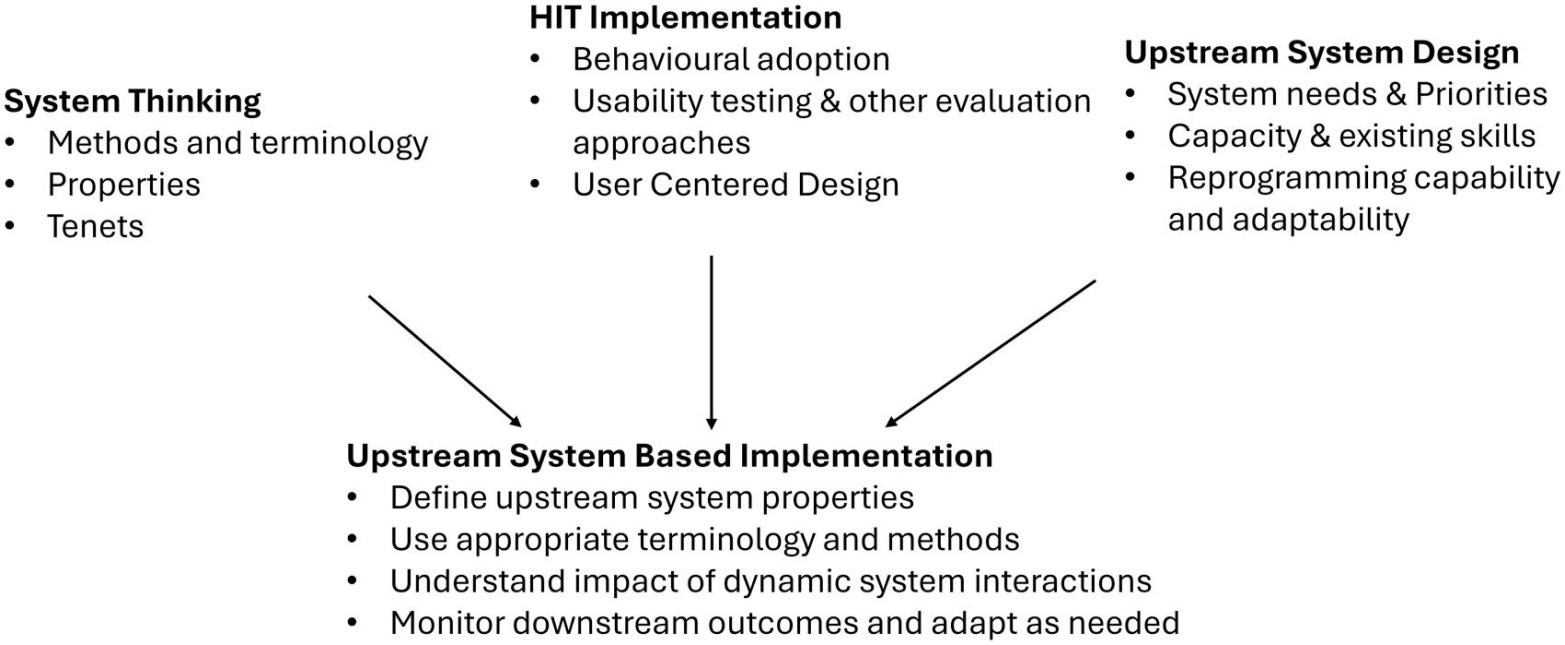
Summary of existing literature and upstream systems-based implementation science approach.

Figure 1 summarizes the above literature into three key themes: systems thinking, HIT implementation, and upstream system design. We then integrate the themes into an upstream systems-based implementation science approach that combines rigor and practicality. Our approach has four main elements: defining upstream properties, using appropriate terminology and methods, understanding dynamic system interactions, and monitoring and responding to downstream outcomes.

### Case example

While BMI research often draws on systems thinking and implementation science, we lack a synthesized framework and pragmatic principles to guide the implementation of HIT. Below, we provide a case example that illustrates the challenges of implementing new technology and how the principles outlined in this paper can support upstream systems-based thinking throughout the entire HIT implementation continuum.

A hospital activated the ambient listening technology features built into its electronic health record (EHR) system shortly after they became available, enabling their use across the organization. Some uses include scribing in the outpatient setting, such as provider documentation and identifying patient instructions, as well as ambient listening during surgical procedures for documentation and predicting when the procedure will be completed. This information is used by stakeholders, such as anesthesia, to plan for patient awakening and to inform post-procedure staff and the floor where the patient will be placed, ensuring care is planned effectively. The organization is considering expanding ambient listening to include generative AI to develop patient instructions and physician orders.

With this technology expansion, the organization aims to adopt a proactive approach to minimizing UICs across the entire implementation continuum. Table 1 extends Figure 1 by developing a four-stage systems-based implementation approach. The case example illustrates how the approach would be used.

**Table 1.** Four-stage systems-based implementation approach

Implementation considerations by stage	Applying upstream systems-based approaches	Case example application
Stage 1—Map out core upstream systems properties, including organizational resilience factors	Identify system properties and stakeholder groups that will be directly and indirectly affected by the changes (eg, technology users, patients/families, people impacted by process changes) and core resilience factors (e.g, goal goal-focused solution and planning and adaptive capacity^35^) that could influence downstream system changes Study and map existing workflow processes to understand how changes will impact system components Determine internal and external system components that could impact proposed changes (eg, policy/legal, organizational, payor mandates, quality metrics)	Conduct field observations and interviews focused on how orders are currently entered into the system and how patient education materials are currently developed and distributed Develop system process and component maps to understand how system properties will change in moving downstream Interview patient education staff and patients/families to ensure the overall system is well understood
Stage 2—Involve Stakeholders throughout decision-making, design, and implementation	Use appropriate methods and approaches to understand perceived changes to system components and how different groups will be impacted Develop communication plans to ensure consistent terminology use and clear messages about project plans	Use approaches such as focus groups with stakeholders to discuss upcoming changes. Trade-offs may have to be negotiated across different groups Distribute information and education materials across suitable channels about the new features
Stage 3—Plan for sustainability	Ensure relevant changes are adopted with full use of new features across micro, meso, and macro levels Identify resources needed to scale from pilot level to full organizational scale-up, realizing redesign may be needed to mitigate UICs Determine necessary support levels for sustained success	Evaluate the adoption of the new features, focusing on dynamic changes to workflow and other systems factors, and the emergence of new errors and unintended consequences Reinfuse as indicated to support system evolution Ensure adequate support staff are available to address any issues
Stage 4—Ongoing evaluation and continuous improvement	Identify downstream system-based metrics to track success and proactively identify unintended consequences Enable incorporation of generative AI technology to monitor metrics in a Learning Health System approach to support ongoing organizational improvement Monitor the above metrics and adapt as needed to any issues identified through ongoing evaluation	Take a “closed loop” approach to evaluation by using feedback to guide future system changes or new features Circle back to stage 1 core systems properties and resilience factors to ensure they are reset for the evolved system and that adaptive capacity is continually monitored going forward.

### Calls to action

Evidence-based principles, utilizing systems thinking and implementation science in BMI and related disciplines, are often underutilized in favor of developing new theories or are abandoned altogether due to time, budget, or other constraints. Integrating systems thinking and implementation science can support upstream approaches that adapt to changing organizational, technical, political, and environmental landscapes over the entire upstream-downstream implementation continuum.

Table 1 presents a four-stage systems-based implementation approach. We summarize the perspective presented in this paper with five pragmatic principles to support upstream systems-based implementation research.

1. Components such as users, policies, technology, organizational, and social factors are all systems. Systems analysis and design must view implementation as a system of systems, rather than as individual entities. We cannot assume how system interactions will influence one another. Rather, we must accurately model a system to understand how changing one part of it will impact the overall system.
2. Systems thinking on its own will not address implementation issues. Rather, it serves as a lens to complement other approaches to HIT implementation, such as business process modeling, human factors, and social and organizational approaches. Systems-based approaches visualize a system to help identify where existing evidence could support HIT implementation.
3. Evidence-based approaches must be fit for purpose. We must use appropriate terminology for systems analysis and design. Complexity refers to a system with many interrelated components and significant interaction among them. We need to move beyond simply calling an implementation “complex” without explaining why. Thinking everything is complex also risks over-engineering a solution to the problem we are trying to solve. Similarly, we cannot use the term “implementation” as a one-size-fits-all term for introducing new HIT or other digital health tools into a setting. Implementation is an iterative process that involves understanding a system and aligning its technical, social, organizational, and other components.
4. Systems thinking helps us understand similarities and differences across health systems. We cannot simply compare two health systems without understanding the various components that define a system, including political, economic, social, and other factors. A successful implementation in one setting does not always translate to success in another context.
5. System-based approaches inspire us to think in terms of lifecycles and iterative upstream-downstream approaches. Health systems are invariably a work in progress, and the learning health system paradigm lies at the core of systems-thinking approaches.

## Conclusion

In conclusion, we must stop reinventing the wheel by developing new implementation theories and practices; instead, we should draw on the existing literature regarding evidence-based principles in systems thinking and implementation science. Technologies are constantly evolving, and new features and ideas emerge, but underlying system-based implementation principles remain applicable. This paper provided a four-stage systems-based approach and a set of pragmatic principles to support the Integration of Systems Thinking and Implementation Science in Biomedical Informatics Research. Our findings build on the strong foundation of systems thinking and implementation science research and practice, providing guidance on how to incorporate this knowledge systematically and practically.

## Authors contributions

All authors contributed to the initial conceptualization of the manuscript. Craig Kuziemsky led the design and writing of the original draft and the revised manuscript. All authors contributed to the writing of the original draft and to the review & editing of the manuscript revisions.

## Data Availability

No new data were generated or analysed in support of this research.
